# Generation of Intestinal and Colonic Organoids Derived From Human Pluripotent Stem Cells

**DOI:** 10.1111/boc.70044

**Published:** 2025-12-12

**Authors:** Lola Bonneau, Lisa Brossard, Theo Noël, Victor Perreaux, Laura Bachir, Archie Khan, Simon Vales, Maxime M. Mahe

**Affiliations:** ^1^ Nantes Université, CHU Nantes, Inserm, TENS, The Enteric Nervous System in Gut and Brain Diseases, IMAD Nantes France; ^2^ Center for Stem Cell and Organoid Medicine Cincinnati Children's Hospital Medical Center Cincinnati Ohio USA

**Keywords:** colon, directed differentiation, human pluripotent stem cells, organoid, small intestine

## Abstract

Over the past decade, significant advancements have been made in understanding the developmental mechanisms involved in human gastrointestinal formation, with organoids emerging as key experimental models. These three‐dimensional in vitro cellular structures mimic the organization and functions of various gut regions, providing a powerful tool for research. By replicating critical stages of gut development, we can now direct the differentiation of cells into specific gastrointestinal tissues. In this protocol, we outline how to generate two types of organoids derived from human pluripotent stem cells (hPSCs): human intestinal organoids (HIOs) and human colonic organoids (HCOs). First, we induce definitive endoderm formation to produce these organoids and specify midgut/hindgut tissues. Three‐dimensional spheroids form spontaneously, can be collected, embedded in an extracellular matrix, and cultured over time. During this phase, the organoid epithelium develops, supported by a mesenchymal layer that promotes maturation and differentiation. After a month of culture, HIOs and HCOs reach a developmental and maturation stage comparable to that of the human fetal intestine. These organoids can be used to study human gastrointestinal development, model diseases, and test therapeutic agents.

## Graphical abstract

Human pluripotent stem cells can be guided through a stepwise differentiation process to produce self‐organizing intestinal and colonic organoids. The resulting organoids recapitulate fetal‐stage epithelial and mesenchymal organization, offering a powerful model to explore human gastrointestinal development and its disorders.

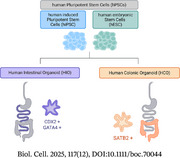

## 1 Introduction

### 1.1 Gut Structure and Function

The gastrointestinal tract is a vital organ that coordinates essential processes, including nutrition, immunity, and the maintenance of overall body homeostasis. This highly organized tubular structure consists of several layers, each contributing to different functions. Beginning with the innermost layer, an epithelial monolayer forms the mucosa—a selective barrier that allows nutrients and water to pass while blocking pathogens. Beneath the epithelium is the muscularis mucosae, a thin layer of smooth muscle that marks the boundary with the submucosa. Within this layer is the submucosal plexus (Meissner's plexus), a component of the enteric nervous system (ENS) that controls secretory functions. Surrounding the digestive tract is the muscularis, which has two layers of smooth muscle: an inner circular layer and an outer longitudinal layer. Between these layers is the myenteric plexus (Auerbach's plexus), another component of the ENS responsible for regulating intestinal motility, including peristalsis and segmentation (Khlevner et al. 2021). In addition to digestion, the gastrointestinal system plays a key role in immune defense, harboring a resident immune system that guards against pathogens. Because of these diverse roles, the digestive tract is highly specialized and divided into several distinct regions, including the small intestine and colon. Along the proximal‐distal axis, the small intestine is divided into the duodenum, jejunum, and ileum, while the large intestine comprises the ascending, transverse, descending, and sigmoid colon. These segments differ anatomically and functionally, with these differences appearing early in embryonic development.

### 1.2 Gut Development

In humans, the gastrointestinal tract begins to develop around the third week of gestation during gastrulation, a critical phase of primary morphogenesis in which the three primitive germ layers form. The outer ectoderm gives rise to the nervous system and skin, while the mesoderm develops into the mesenchyme, an embryonic connective tissue that surround the growing digestive epithelium. Initially made up of fibroblasts, this mesenchyme also contains muscle cells, which later become the intestinal smooth muscles and blood vessels in adults (Chin et al. 2017, Flatres et al. 2019). The innermost layer, the endoderm, differentiates into the intestinal epithelium (Zorn and Wells 2007). By the fourth week of gestation, the endoderm elongates and invaginates to form the primitive gut, which is divided into three regions: the foregut, midgut, and hindgut (Kostouros et al. 2020). The midgut will develop into the small intestine, ascending colon, and the proximal two‐thirds of the transverse colon, while the hindgut forms the rest of the colon and rectum (Kostouros et al. 2020). The organ formation of the gut tube is controlled by the spatial and temporal expression of several genes during development. Major pathways like fibroblast growth factor (FGF) and Wingless‐Type MMTV Integration Site Family (WNT) trigger midgut and hindgut specification and activate the homeobox protein CDX‐2 (CDX2), which plays a key role in epithelial development and intestinal identity (Chin et al. 2017). Recent studies have demonstrated a crucial role of the Bone Morphogenetic Protein (BMP) pathway in colon specification, which induces the expression of posterior homeobox genes, including *HOXA10*, *HOXA11*, *HOXA13*, and *HOXD13*. BMP signaling also promotes the expression of the Special AT‐rich Sequence Binding Protein 2 (SATB2), a homeobox transcription factor recognized as a marker for the colon (Múnera et al. 2017). Although the significant stages of intestinal development are now well understood, many physiological and pathological mechanisms in humans are still poorly understood, and studying them often requires embryonic tissues, which are hard to obtain. This limitation has driven to the development of new models over the past decade, notably human intestinal organoids (HIOs) and human colonic organoids (HCOs).

### 1.3 HIO and HCO: Organoids to Mimic Gut Development

Organoids are three‐dimensional, in vitro multicellular structures that replicate part of an organ's histological architecture and some of its functions. Moreover, these models demonstrate self‐organization and self‐renewal (Almeqdadi et al. 2019). In the case of intestinal organoids, two models exist depending on the type of stem cells used. The first, derived from adult stem cells isolated from intestinal crypts, results in enteroids or colonoids that resemble mature adult epithelial tissue (Sato et al. 2009). The second model, based on human pluripotent stem cells (hPSCs), a category that encompasses both human embryonic stem cells (hESCs) and human induced pluripotent stem cells (hiPSCs), provides an early‐stage developmental model of the intestine (Finkbeiner et al. 2015). Currently, organoids derived from hiPSCs, which are generated by reprogramming adult somatic cells, serve as a powerful tool for studying intestinal development, eliminating the ethical concerns associated with using human embryonic tissue. Here, we describe a method for generating HIOs and HCOs derived from hPSCs, thus applicable to both hESCs and hiPSCs. Nevertheless, only hiPSCs were used in the present work. This process involves sequentially adding cytokines and growth factors to the culture medium at specific concentrations and time points to recreate embryonic intestinal development in vitro. The first step is to induce definitive endoderm by adding Activin A, a member of the TGF‐β family, that activates the Nodal pathway and promotes the expression of endodermal markers, such as SRY‐Box Transcription Factor 17 (*SOX17*) and Forkhead Box A2 (*FOXA2*) (D'Amour et al. 2005). Meanwhile, a small subset of cells (∼2%) expresses the mesodermal marker T or Brachyury protein (Spence et al. 2011). These residual cells give rise to the mesenchyme that develops around the epithelial structures of the organoids during their growth and development. Subsequently, activation of the FGF and WNT pathways, thanks to FGF4 and Chiron addition, specifies the midgut identity, leading to the formation of spheroids before yielding HIOs. Finally, the additional activation of the BMP pathway gives HCOs their colonic identity (Múnera et al. 2017). After 28 days of culture in Matrigel, both HIOs and HCOs derived from hPSCs form a monolayer of epithelial cells surrounded by mesenchymal tissue, exhibiting diverse cell types, including enterocytes, Paneth cells, goblet cells, and enteroendocrine cells (Spence et al. 2011) (Figure 1).

### 1.4 Comparisons With Other Models

Compared with traditional animal models, such as mice, HIOs and HCOs provide a more accurate representation of human tissue structure and cellular composition, thereby reducing species‐specific differences in physiology and gene expression (Finkbeiner et al. 2015, Watson et al. 2014). HIOs and HCOs also have several advantages over enteroids and colonoids derived from adult intestinal stem cells isolated from crypts in primary human tissues. Since these enteroids and colonoids primarily consist of epithelial cells, they lack the broader cellular diversity observed in hPSC‐derived organoids, which also contain mesenchymal components. This additional complexity makes hPSC‐derived organoids more suitable for studying developmental processes and cell‐cell interactions (Sato et al. 2009, VanDussen et al. 2015). Organ‐on‐chip platforms excel at mimicking dynamic physiological conditions, such as flow and mechanical stretching, which are challenging to reproduce in static cultures. These systems can complement organoid models by enabling real‐time monitoring and precise environmental control, making them ideal for research on barrier function and drug absorption (Kasendra et al. 2018, Moerkens et al. 2024). When combined, organoids provide the complex tissue architecture and multicellular organization, while organ‐on‐chip systems offer a physiologically relevant microenvironment.

### 1.5 Advantages and Applications

HIOs and HCOs derived from hPSCs offer numerous applications for studying gastrointestinal development, physiology, and diseases, as well as for advancing therapeutic research with translational and preclinical relevance (Xiang et al. 2024). In fundamental research, they serve as valuable tools for developing more complex models to address a range of scientific questions. One potential application involves their transplantation into the kidney capsule of immunocompromised mice, allowing vascularization by the host's circulatory system. This in vivo transplantation promotes tissue maturation and the formation of a more complex architecture, including crypts and villi, thereby overcoming some of the limitations associated with in vitro culture, where achieving complete tissue differentiation can be challenging (Watson et al. 2014). Another key advantage of these models is their ability to be combined with other cell types, thereby increasing complexity and better replicating the in vivo environment. For instance, hPSCs can be differentiated into vagal neural crest cells (vNCCs), which, when co‐cultured with HIOs, integrate into the mesenchyme and contribute to the development of a functional ENS (Workman et al. 2017). Similarly, the incorporation of immune cells such as lymphocytes and macrophages can further enhance organoid maturation and physiological relevance (Song et al. 2024). Intestinal organoids are also particularly useful for studying a variety of intestinal pathologies, including congenital disorders such as Hirschsprung's disease and pediatric intestinal pseudo‐obstruction (PIPO). Genome‐editing tools such as CRISPR‐Cas9 can be employed to introduce specific mutations, enabling the modeling of genetic diseases and providing insights into their underlying mechanisms (Balmas et al. 2023, Sanchez et al. 2024). Ultimately, organoids also hold a significant potential for therapeutic development. Organoids derived from patient‐specific hiPSCs can be used in translational studies to evaluate drug responses in a personalized medicine approach. In this context, patient‐derived colorectal organoids (tumoroids) have been increasingly employed to model tumor heterogeneity and assess responses to standard and experimental chemotherapeutic agents. These systems enable high‐throughput drug screening and provide a powerful platform to predict patient‐specific sensitivity or resistance to treatment, thereby contributing to precision oncology (Cartry et al. 2023; Mao et al. 2024). Patient‐derived organoid models thus provide a robust platform for testing both existing and novel drug candidates, with an increasing number of clinical trials now highlighting their translational relevance in gastrointestinal disease therapy.

### 1.6 Limitations

Despite their significant potential and broad applications, HIOs and HCOs face several limitations that need to be considered. For example, a major limitation is the lack of vascularization, which restricts oxygen supply and consequently limits tissue growth and functional maturation. However, some strategies to address these limitations have been discussed previously, including in vivo transplantation into animal recipients to promote vascular integration. On the technical side, maintaining and differentiating hPSCs requires specialized expertise and is time‐consuming, making large‐scale production difficult. Additionally, directed differentiation protocols often show high variability. Differences in the number of spheroids produced, the epithelial‐to‐mesenchymal ratio, and gene expression profiles across batches reduce the reproducibility of experiments (Holloway et al. 2019). Despite these challenges, HIOs and HCOs remain powerful and highly relevant models that greatly improve our understanding of human gastrointestinal development and disease mechanisms.

## 2 Timing

**FIGURE 1:**
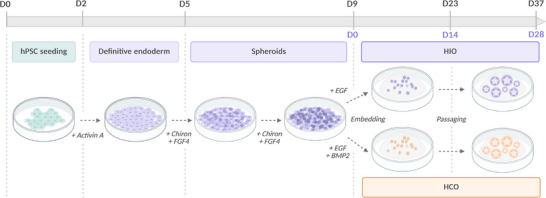
Overview of the protocol steps. The complete directed differentiation process lasts for 37 days, including 28 days of culture in Matrigel. Two days after seeding the hPSCs, the first step involves adding Activin A for 3 days to generate definitive endoderm formation, followed by the addition of FGF4 and CHIRON for 4 more days to induce spheroid formation. The resulting spheroids are then embedded in Matrigel and cultured for 28 days, with a passage at day 14 to renew the Matrigel.

## 3 Materials

**Biological materials**

- hPSC lines: hESCs or hiPSCs.

**▲CRITICAL**. Short tandem repeat (STR) profiling should be performed on all hPSC lines. Furthermore, routine karyotyping and regular mycoplasma testing are essential to maintain genomic stability and prevent hidden contamination.
**▲CRITICAL**. The quality of hPSC lines used should be routinely verified by characterizing their pluripotency and monitoring their ability to differentiate into endodermal, mesodermal, and ectodermal lineages. The cell line used in this study is an hiPSC line, LON71.019 (hiN.FM.m.LON71.019 from the iPS Core Facility of Nantes).
**▲CRITICAL**. Consult with your institution to ensure compliance with national and institutional guidelines and regulations. Appropriate consent procedures and administrative rules must be adhered to for working with hESCs and hiPSCs.

**Cell line quality control assays**

- Cell line identity: Cell line authentication by short tandem repeat (STR) analysis, provided as a service by Stem Genomics.
- Genomic stability:
- Routine quality control during amplification and maintenance: iCS‐digital PSC – 28‐probe test to detect the most recurrent genetic abnormalities, provided as a service by Stem Genomics.
- Exhaustive quality control at cell line reception and/or at the end of banking:
- Duo iCS‐Karyo, provided as a service by Stem Genomics.
- Molecular karyotyping, provided as a service by Life & Brain GmbH.
- Mycoplasma detection:
- Mycoplasma Detection Kit (MycoStrip 100, InvivoGen, cat. no. rep‐mysnc‐100)

**NOTE**: A thermocycler is required to perform the mycoplasma detection assay using this kit.

**Reagents**

- mTeSR1 (mTeSR 1, Stem Cell Technologies, cat. no. 85850)
- CS10 (CryoStor CS10, Stem Cell Technologies, cat. no. 100‐1061)
- XF solution (StemMACS Passaging Solution XF, Miltenyi Biotec, cat. no. 130‐104‐688)
- Dispase (5 U/mL dispase in Hanks' Balanced Salt Solution, Stem Cell Technologies, cat. no.07913)
- DPBS ‐/‐ 1X (Dulbecco's Phosphate Buffered Saline without Calcium and Magnesium, Eurobio, cat. no. CS1PBS0101)
- ROCKi (ROCK specific inhibitor, Y27632, StemCell Technologies, cat. no. 72304)
- DMEM/F12 (Gibco Dulbecco's Modified Eagle Medium, F12 nutrients, HEPES, Life Technologies, cat. no. 31330038)
- RPMI Gibco (Roswell Park Memorial Institute Medium 1640, Glutamine, Life Technologies, cat. no. 21875034)
- Accutase (ACCUTASE, StemCell Technologies, cat. no. 07920)
- Act. A (Human recombinant Activin A premium grade, Miltenyi Biotec, cat. no. 130‐115‐011).
- **▲CRITICAL**. Stock aliquots should be stored at –80°C and prepared in a suitable volume to minimize multiple freeze‐thaw cycles. Aliquots should be kept on ice at 4°C when used to avoid multiple freeze/thaw cycles.
- FGF4 (Human recombinant fibroblast growth factor 4 premium grade, Miltenyi Biotec, cat. no. 130‐109‐391)

**▲CRITICAL**. Stock aliquots should be stored at –80°C and prepared in a suitable volume to minimize multiple freeze‐thaw cycles. Aliquots should be kept on ice at 4°C when used.

- Chiron (StemMACS CHIR99021 in Solution, Miltenyi Biotec, cat. no. 130‐106‐539)
- **▲CRITICAL**. Stock aliquots should be stored at –80°C and made in a suitable volume, avoiding multiple freeze/thaw cycles. Aliquots should be kept on ice at 4°C when used.
- MEM NEAA (Gibco Minimum Essential Medium Non‐Essential Amino Acids 100x, Life Technologies, cat. no. 11140035)
- HiFBS (Gibco Fetal Bovine Serum, certified, heat‐inactivated, United States, Life Technologies, cat. no. A3840001)
- Matrigel GFR (Matrigel Growth Factor Reduce basement membrane matrix phenol‐red free, Corning, cat. no. 356231).

**▲CRITICAL**. Stock aliquots should be stored at –80°C and prepared in a suitable volume to minimize multiple freeze‐thaw cycles. Always keep Matrigel on ice until coating.

- Matrigel ES (Matrigel hESC‐Qualified Matrix, Corning, cat. no. 354277)

**▲CRITICAL**. Stock aliquots should be stored at –80°C and prepared in a suitable volume to minimize multiple freeze‐thaw cycles. Always keep Matrigel on ice until coating.

- hEGF (Human recombinant epithelial growth factor, premium grade, Miltenyi Biotec, cat. no. 130‐097‐751).

**▲CRITICAL**. Stock aliquots should be stored at –80°C and made in a suitable volume, avoiding multiple freeze/thaw cycles. Aliquots should be kept on ice at 4°C when used.

- BMP2 (Human recombinant bone morphogenetic protein 2 premium grade, Miltenyi Biotec, cat. no. 130‐110‐926).

**▲CRITICAL**. Stock aliquots should be stored at –80°C and made in a suitable volume, avoiding multiple freeze/thaw cycles. Aliquots should be kept on ice at 4°C when used.

- Ad DMEM (Gibco Advanced Dulbecco's Modified Eagle Medium, F12 nutrients, Non‐Essential Amino Acids, Sodium Pyruvate, Life Technologies, cat. no. 12634010)
- Glutamax (Gibco GlutaMAX Supplement 100x, L‐alanyl‐L‐glutamine, Life technologies, cat. no. 35050038)
- Hepes (Gibco acide sulfonique N‐2‐hydroxyéthylpiperazine‐N‐2‐éthane, Life technologies, cat. no. 15630056)
- Pen/Strep (Penicillin/Streptomycin, Eurobio cat. no. CABPES01‐0U)
- N2 supplement (Gibco, Life Technologies, cat. no. 17502048)
- B27 supplement (Gibco, Life Technologies, cat. no. 17504044)
- Cell Recovery (Corning, Cell Recovery Solution, 100 mL, cat. no. 354253)
- BSA (Bovine Serum Albumine solution, Merck, cat. no. A9576)

**Equipment**

- Horizontal laminar flow hood
- Cell culture centrifuge (ThermoFisher, Heraeus Megafuge 8R)
- Inverted microscope (Leica DMi1)
- CO_2_ incubator with a controlling and monitoring system for CO_2_, humidity, and temperature
- Refrigerator at 4°C, and freezers at –20°C and –80°C
- Hemocytometer (Malassez 0.2 mm counting chamber, Marienfeld Superior cat. no. 0640610)
- 3D stereoscopic microscope under horizontal laminar flow hood (Lynx Evo‐Biomed with Ergo Slim Inlus Statif, Vision Engineering, cat. no. EVO501_BIOMDV587631)

**NOTE**: These tools are not necessary if you use method B for passaging organoids.

- qPCR system (StepOnePlus Real‐Time PCR System, Life Technologies, cat. no. 4376600)

**Cell culture disposables**

- Petri dishes, multiwell plates, conical tubes, pipettes, pipette tips, cell scrapers, and so on.
- Microdissection tools:
- 1 x Curved forcep (Dumont #7 Forceps—Biology Tips/Curved/Dumostar/ 11.5 cm, F.S.T, cat. no. 11297‐10)
- 2 x Straight forceps (Dumont #5 Fine Forceps—Biology Tips/Straight/Inox/ 11 cm, F.S.T, cat. no. 11254‐20)
- 1 x Spring straight scissors (Vannas Spring Scissors—5 mm Cutting Edge, F.S.T, cat. no. 15044‐08)

**NOTE**: These tools are not necessary if you use method B of passaging organoids.

- Tissue culture‐treated 6‐well plate (Corning, cat. no. 3516)
- Tissue culture‐treated 24‐well plate (Corning, cat. no. 3524)
- Nunc‐treated 24‐well plate (Nunc Cell Culture Processed Multiwell Dishes, Life Technologies, cat. no. 142475)
- Conical tubes 15 mL (Sarstedt, cat. no. 62.554.502)
- Conical tubes 50 mL (Sarstedt, cat. no. 62.547.254)
- CoolCell freezer (CoolCell Freezing Containers, Corning, cat. no. 432000)
- Cryogenic vials 2 mL (Corning, cat. no. 431386)

**Reagent setup**

▲ **CRITICAL**. Concentrations listed below are final concentrations. Final and stock concentrations can be found in Tables 1, 2, and 3.
▲ **CRITICAL**. All solutions, including stock solutions, should be prepared under sterile conditions in a laminar flow hood to avoid contamination.
▲ **CRITICAL**. Avoid prolonged exposure to the light of the culture media.

**TABLE 1:** List of growth factors and small molecules.

Recombinant protein per small molecule	Stock Concentration	Dilution	Final Concentration	Company	Cat. No.
ROCKi	10 mM	1 :1000	10 µM	StemCell Tech.	72304
Act. A	100 µg/mL	1 :1000	100 ng/mL	Miltenyi Biotec	130‐115‐011
FGF4	100 µg/mL	1 :200	500 ng/mL	Miltenyi Biotec	130‐109‐391
CHIR99021	10 mM	1 :3000	3 µM	Miltenyi Biotec	130‐106‐539
EGF	500 µg/mL	1 :5000	100 ng/mL	Miltenyi Biotec	130‐097‐751
BMP2	100 µg/mL	1 :1000	100 ng/mL	Miltenyi Biotec	130‐110‐926

**TABLE 2:** List of matrix for hPSCs and organoids.

Matrix	Stock concentration	Dilution	Final concentration	Company	Cat. No.
Matrigel GFR	Around 8–10 mg/mL (depending on batch)	1 :1	Around 8–10 mg/mL (depending on batch)	Corning	356231
Matrigel ES	Not known as per manufacturer's product information	Around 1 :100 (depending on batch)	Not known	Corning	354277

**TABLE 3:** List of products and recipe for Minigut medium.

Product	Stock concentration	Dilution	Final concentration	Company	Cat. No.
Ad DMEM	Depending on batch	1 :1	Depending on batch	Life tech.	12634010
Glutamax	200 mM	1 :100	2 mM	Life tech.	35050038
Hepes	1 M	1 :100	10 mM	Life tech.	15630056
N2 supp	Depending on batch	1 :100	Depending on batch	Life tech.	17502048
B27 supp	Depending on batch	1 :50	Depending on batch	Life tech.	17504044
Pen/Strep	10,000 U/mL 10,000 g/mL	1 :100	100U/mL 100 g/mL	Eurobio	CABPES01‐0U

**Activin A (AA) Media: for endoderm induction, Day 2 to Day 4**

▲ **CRITICAL**. Prepare the differentiation media extemporaneously and add cytokines just before the media change.

**Day 1: AA1**. Combine RPMI 12.857 mL with 130 µL MEM NEAA 100x. Add 13 µL of 100 µg/mL Act. A.
**Day 2: AA2**. Combine RPMI 12.831 mL, with 130 µL MEM NEAA 100x, with 26 µL HiFBS. Add 13 µL of 100 µg/mL Act. A.
**Day 3: AA3**. Combine RPMI 12.6 mL, with 130 µL MEM NEAA 100x, with 260 µL HiFBS. Add 13 µL of 100 µg/mL Act. A.

**FGF4/CHIR (FC) Medium: for midgut/hindgut regionalization, Day 5 to Day 8**

Combine RPMI 12.6 mL, with 130 µL MEM NEAA 100x, with 260 µL HiFBS. Add 65 µL of 100 µg/mL FGF4 and 3.9 µL of 10 mM CHIR99021.

**Minigut Medium: For organoids's growth Day 9 to Day 37**

Combine 470 mL of Ad DMEM with 5 mL of N2 supplement, 10 mL of B27 supplement, 5 mL of 100x Pen/Strep, 5 mL of 100x Glutamax, and 5 mL of 100x Hepes. Add EGF extemporaneously at a final concentration of 100 ng/mL to prepare the complete Minigut medium.

**▲CRITICAL**. Stock aliquots should be stored at –80°C and made in a suitable volume, avoiding multiple freeze/thaw cycles. Aliquots should be kept on ice at 4°C when used.

**Minigut B Medium: For colonic regionalization Day 9 to Day 12**

Dilute BMP2 at 100 ng/mL in complete Minigut medium (with EGF).

**Matrigel ES**

Thaw a frozen vial of Matrigel ES overnight at 4°C. Prepare approximately 220 µL aliquots in 1.5 mL tubes (depending on the batch, refer to the batch data sheet). Use chilled pipette tips to prepare the aliquots and store them frozen at −20°C.

▲CRITICAL. Matrigel must be kept cold to prevent polymerization.

**Equipment setup**

**▲CRITICAL**. All reagents should be prepared under sterile conditions in a laminar flow hood to avoid contamination.

**Matrigel ES‐coated plates for use in Steps 1, 10, and 22**.

Thaw an aliquot of Matrigel ES on ice 1 h before dilution. Dilute the aliquot of Matrigel ES in the recommended volume of cold DMEM:F12 (see manufacturer's certification sheet). Coat the wells with the diluted Matrigel solution (1 mL per well of a 6‐well plate or 300 µL per well of a 24‐well plate) and let them incubate at 37°C for 2 to 48 h. Aspirate the Matrigel solution before plating hPSCs.

**▲CRITICAL**. To achieve a fully defined system, substitute Matrigel‐coated plates with vitronectin‐coated plates.

## 4 Procedures

**Procedure 1: hPCSs cultures**

**Thawing frozen hPSCs**

Maintaining high‐quality hPSCs is essential for successful differentiation and efficient production of HIOs and HCOs. Although various hPSC cell lines can be used, each may behave differently in culture, for instance, in terms of cell growth. Culture conditions, including maintenance, frequency of passaging, and cell density at seeding prior to differentiation, may need to be optimized accordingly.

**▲CRITICAL**. Frozen stocks of hPSCs should be kept in a liquid‐nitrogen cryogenic storage system at −156°C. Frozen cells should be thawed and plated immediately.

**Maintaining hPSC cultures: Feeder‐free method**

**▲CRITICAL**. mTeSR1 medium should be used at room temperature and should not be left out of the refrigerator for more than 30 min.

1. Coat two wells of a 6‐well plate with diluted Matrigel ES in DMEM:F12 as described above before thawing.
2. Remove a vial of hPSCs from liquid nitrogen and gently warm it in your hands.

**! CAUTION** Wear cryogenic gloves and safety glasses when handling liquid nitrogen. Ensure the room is well ventilated, and keep the window open.

1. When only a small pellet of ice remains, spray the vial with 70% ethanol (diluted with H2O, vol/vol), and transfer it to a laminar flow hood.
2. Add 1 mL of mTeSR1 at room temperature (20°C–25°C) directly into the vial and gently mix by pipetting up and down one to three times.
3. Transfer the cell suspension to a conical tube.
4. Adjust the volume to 5 mL of mTeSR1 for 500 µL of initial frozen suspension.
5. Centrifuge the conical tube at 140 x *g* for 5 min at room temperature (20°C–25°C).
6. Carefully aspirate the supernatant, avoiding contact with or aspiration of the pellet.
7. Gently resuspend the pellet in 1 mL of mTeSR1 supplemented with 10 µM ROCKi.
8. Aspirate the Matrigel solution from the coated plates and replace it with 1.5 mL of mTeSR + 10 µM ROCKi.
9. Plate the cell clumps in suspension into two coated wells (333.3 µL of suspension into one well and 666.66 µL into the other).
10. Incubate the plated cells in a cell culture incubator at 37°C and 5% CO_2_.
11. Replace the medium daily with mTESR1 without ROCKi. When the cells reach confluency, expand the colonies as described below.

**Maintaining hPSCs cultures**

**▲CRITICAL**. It is essential to be proficient in maintaining hPSCs cultures before using them for endoderm induction and midgut/hindgut organoid formation. Newly thawed cell lines should be passaged at least twice before initiating differentiation using the protocol of your choice, such as the one described below.

**NOTE**: Alternative methods, such as enzymatic single‐cell dissociation, can also be employed for passaging hPSCs. However, the mechanical method described here offers the advantage of maintaining high cell viability. When passaged as small cell clumps, intercellular junctions are preserved, thereby reducing cellular stress and supporting the maintenance of pluripotency. Nevertheless, single‐cell dissociation allows precise control of seeding density, whereas the mechanical method results in heterogeneous clumps of variable size.

1. Passage hPSCs using the procedure below.
2. **▲CRITICAL**. hPSCs should be passaged at least once every 5–7 days, when they reach approximately ∼ 70%–80% of confluency.
3. Coat the required number of wells in a 6‐well plate with 1 mL of diluted Matrigel ES between 2 and 48 h before splitting, and keep the plate in a cell culture incubator at 37°C and 5% CO_2_. On the day of splitting, check for differentiated cells as described below.
4. Aspirate the mTeSR1 medium and wash each well with 1 mL of PBS, gently rocking the plate before aspirating the PBS.
5. Add 1 mL of XF passaging solution to each well and incubate for 3 min at room temperature.
6. During incubation, replace the Matrigel ES solution on coated plates with 1.5 mL of mTeSR1.
7. Aspirate XF and replace it with 1 mL of mTeSR1.
8. Scrape the colonies using a sterile 200 µL filter tip. Move the tip from right to left (10–12 times), then from left to right (10–12 times), followed by from top to bottom (10–12 times), and from bottom to top (10–12 times). Finally, scrape around the well by moving the tip in a clockwise direction from 12 o'clock to 12 o'clock, then counterclockwise starting at the corner of the well.

**NOTE**: Colonies will be cut into even squares.

- Gently pipette the corresponding volume of homogeneous cell clumps solution to achieve a splitting ratio between 1:6 and 1:10 (166.6 µL to 100 µL).

**NOTE**: A ratio of 1:6 is best for maintaining optimal hPSCs quality.

- Gently distribute cell clumps into the appropriate pre‐coated wells containing mTeSR1.
- Gently swirl the plate in a circular motion to homogenize the cell distribution, then incubate in a cell culture incubator at 37°C with 5% CO_2_.
- Change the cell culture medium daily and monitor spontaneous differentiation as described below.
- Check hPSC colonies under an inverted microscope.
- Mark differentiated zones using a non‐permanent marker.

**FIGURE 2:**
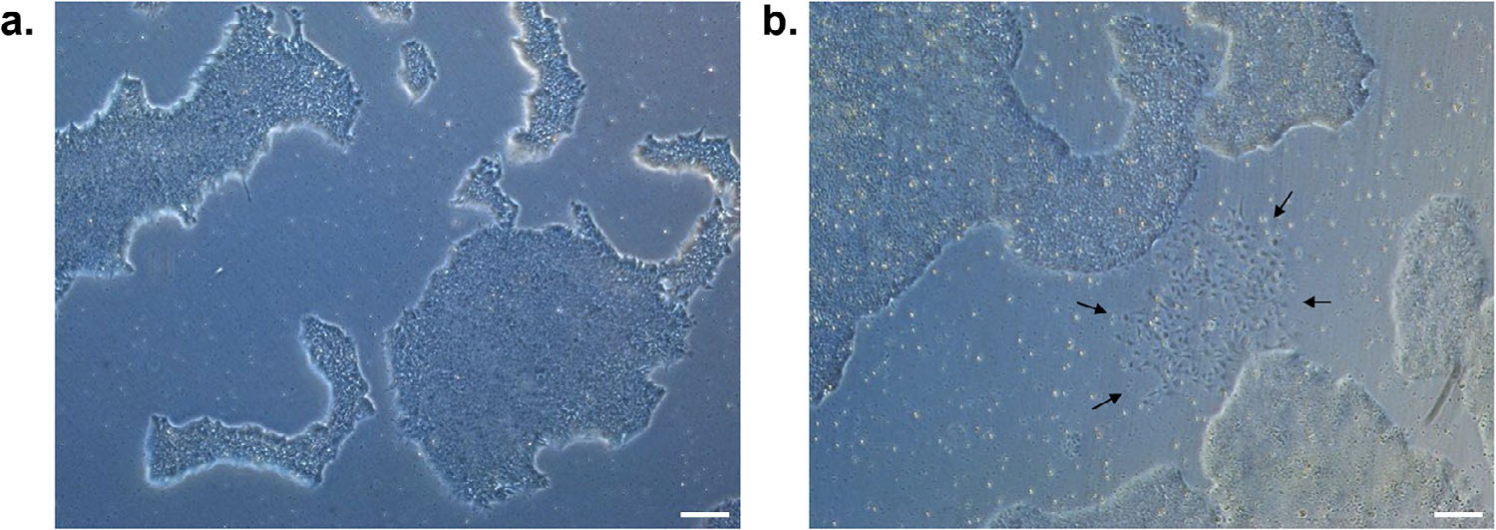
Representative cultures of LON71.019 hiPSCs during maintenance.

**▲CRITICAL**. Differentiated cells must be checked 2 days after passaging and every day thereafter. Pluripotent colonies contain cells with a defined nucleus; they are homogeneous and display well‐defined borders. In contrast, differentiated colonies either consist of large cells without clear colony borders or exhibit a heterogeneous zone at the colony center, with occasional three‐dimensional structures emerging from the center (Figure 2).

- Aspirate the mTeSR1 medium and marked areas from the culture, then replace with fresh mTeSR1 at room temperature.
- Incubate the plate in a cell culture incubator at 37°C with 5% CO_2_.

**Freezing hPSC cultures**

1. Wash the culture with 1 mL of PBS per well.
2. Add 1 mL of dispase to each well and incubate at 37°C with 5% CO_2_ for 6–7 min.

**NOTE**: Dispase should be at 1 U/mL diluted in DMEM/F12.
When the edges of colonies appear refractive under an inverted microscope, aspirate the dispase and wash the wells with 1 mL of DMEM/F12.
**▲CRITICAL**. This step is crucial to completely remove dispase.
Add 1 mL of mTeSR1 per well and scrape the colonies using a sterile 1000 µL filter tip. Use a disinfected pipette to hold the filter tip. Scrape the well by moving the tip from right to left (10–12 times), then from left to right (10–12 times), followed by from top to bottom (10–12 times), and finally from bottom to top (10–12 times). Finish by scraping around the well in a clockwise direction from 12 o'clock to 12 o'clock, then counterclockwise starting from the corner of the well.
**NOTE**: Colonies will be divided into even squares.

1. Transfer the scraped cells into a 15 mL conical tube.

**▲CRITICAL**. Do not exceed three wells per tube to avoid pellet adhesion and minimize cell death.

1. Add 1 mL of mTeSR1 per well and flush the wells to ensure all cells are collected, then transfer to the corresponding tubes.
2. Centrifuge the tubes for 5 min at 140 x *g* at room temperature. Discard supernatant and resuspend the pellet delicately with an appropriate volume of cold CS10 (500 µL of CS10 per well).
3. Transfer 500 µL of the cell suspension into each pre‐labeled cryovial. Place the vials in a CoolCell freezing container and freeze directly at −80°C.
4. Transfer the vials to a liquid nitrogen cryogenic storage system at −156°C within 80 min to 48 h, and update the cell bank records in your laboratory notebook.

**Procedure 2: Directed differentiation**

**hPSC seeding (Day 0–1) and endoderm induction (Day 2–4)**

1. Coat the required number of wells in a 24‐well plate with diluted Matrigel ES (300 µL per well) between 2 and 48 h before plating, then keep them in a cell culture incubator at 37°C with 5% CO_2_.
2. Choose two hPSC wells at 70%–80% confluency to seed a full 24‐well plate.

**NOTE**: You can also use less confluent cells if they have been growing for 3–4 days; in that case, increase the number of wells used.

1. Prepare mTeSR1 + 10 µM ROCKi (15 mL to fill a 24‐well plate) and DMEM/F12 + 10 µM ROCKi (2 mL per well of a 6‐well plate).
2. Check for differentiated cells (see Step 10.b.) (Figure 2).
3. Aspirate the mTeSR1 medium and the marked areas from the culture wells and, then wash with DMEMF12 + 10 µM ROCKi (1 mL per well).
4. Add 1 mL of Accutase per well and incubate for 5–6 min in the incubator at 37°C with 5% CO_2_.
5. Ensure most cells are detached. If not, incubate for an additional minute, before proceeding.
6. Dilute the Accutase with 1 mL of DMEM/F12 + 10 µM ROCKi per well, gently flush to obtain a single‐cell suspension, and transfer into a 15 mL conical tube.
7. Centrifuge for 1 min at 300 x *g* at room temperature. Discard the supernatant and gently resuspend the pellet in 1 mL of mTeSR1 + 10 µM ROCKi.
8. Transfer 2.4 million cells into a new conical tube and add mTeSR1 + 10 µM ROCKi to reach a final volume of 12 mL.
9. Aspirate the Matrigel ES solution from the coated plates.
10. Plate the cell suspension at approximately ∼53,000 cells cm^−2^ into pre‐coated wells.

**▲CRITICAL**. Handle gently throughout the distribution process. Homogenize by moving back and forth between wells to ensure an even distribution of cells.

**FIGURE 3:**
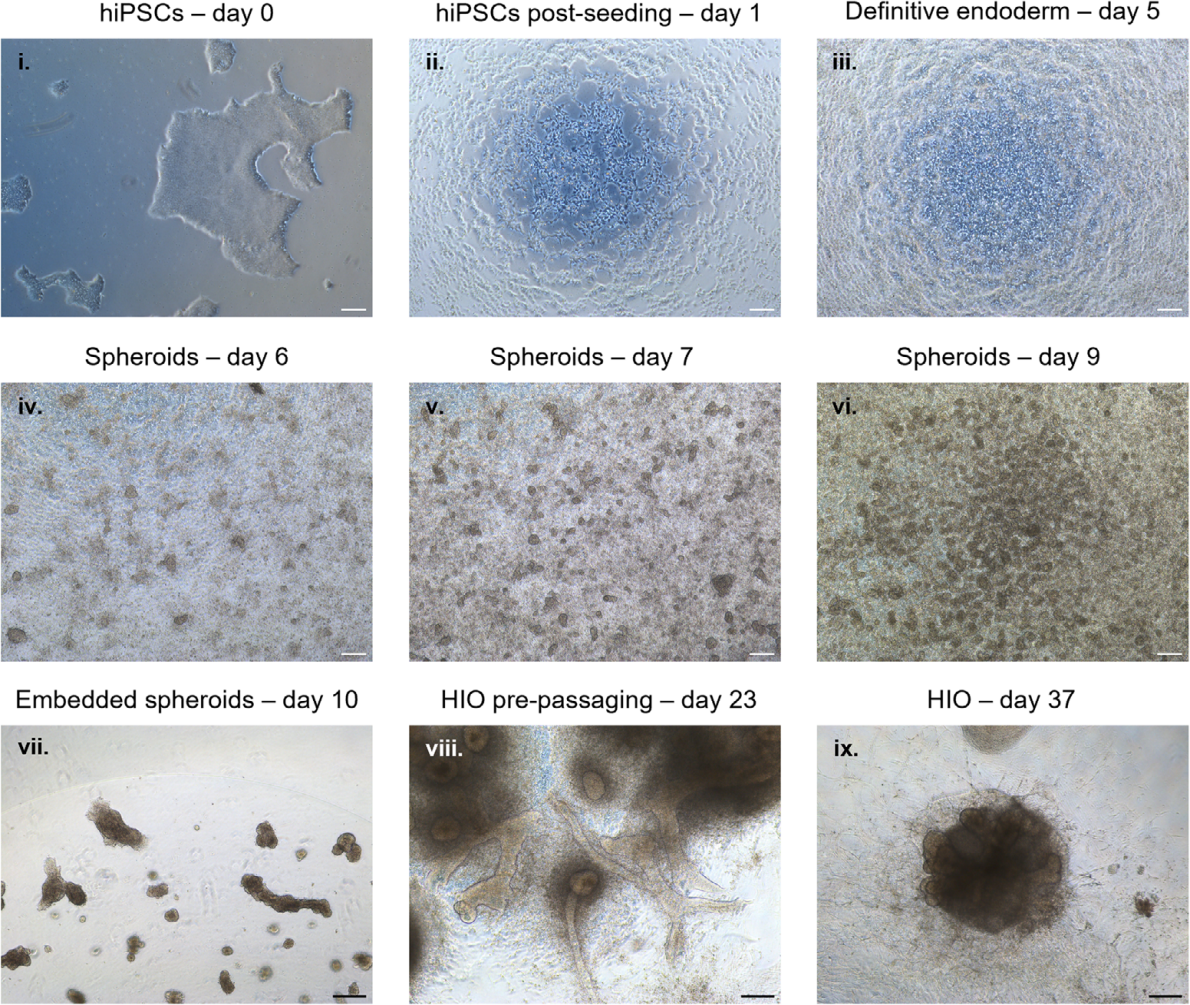
Key stages of differentiation of hiPSCs into intestinal organoids. Representative brightfield images showing different timepoints of directed differentiation from (i) Day 0 (LON71.019 hiPSCs before seeding) (ix) to Day 37 (HIO at the end of culture) under the current differentiation protocol. Scale bars = 200 µm.

1. Gently move the plate to homogenize the cell suspension and incubate at 37°C with 5% CO_2_ for 24 h.
2. Aspirate the mTeSR1 + ROCKi and add an appropriate volume of fresh mTeSR1 (Figure 3ii).
3. Incubate at 37°C with 5% CO_2_ for an additional 24 h.
4. Remove mTeSR1 and add AA1 medium for 24 h. Replace sequentially with AA2 and AA3 media over the following 2 days, respectively (Figure 3iii).

**Midgut/Hindgut regionalization (Day 5–8)**

1. Renew the medium with FC medium daily for four consecutive days (Figure 3iv,v).

**NOTE**: Spheroids generally appear around Day 7 (depending on the hPSC line).
**▲CRITICAL**. Make sure not to aspirate any nascent spheroids that are beginning to detach. From this stage onwards, you may gently remove half of the medium each day with a pipette until embedding. Alternatively, collect all the old medium into a tube and allow the spheroids to sediment before transferring the spheroids into fresh medium.

**Procedure 3: 3D intestinal and colonic organoid cultures**

**Spheroids embedding (Day 9)**

On Day 9, collect the spheroids to embed them in 3D Matrigel culture (Figure 3vi).

1. Thaw the GFR Matrigel at least 2 h on ice before plating.
2. Gently flush the wells to detach the spheroids and collect them using a 1 mL pipette.
3. **▲CRITICAL**. Take care not to detach or collect cells from the adherent cell layer.
4. Pool the collected spheroids in a 15 mL conical tube.

**NOTE**: To increase experimental reproducibility, estimate the spheroid concentration beforehand. To do this, take 20 µL of the well‐homogenized suspension and place it in a Petri dish to count the number of spheroids under a microscope. If the spheroid density is too high for accurate counting, perform a dilution and recount. Then, transfer to a new tube the volume of suspension required to obtain 5–25 spheroids per Matrigel drop, and allow the spheroids to sediment.

1. Place the tube in the incubator at 37°C with 5% CO_2_ for 15 to 30 min to allow sedimentation, or centrifuge for 2 min at 50 x *g* at room temperature.
2. Carefully aspirate the supernatant.
3. On ice, gently resuspend the pelleted spheroids in cold GFR Matrigel (30–40 µL multiplied by the number of drops required). Avoid creating bubbles when mixing.

**▲CRITICAL**. Always use Matrigel on ice to avoid premature polymerization.

1. Deposit 30–40 µL of Matrigel containing spheroids in the center of each well.

**NOTE**: Using Nunc Delta surface‐treated plates at this stage and throughout organoid cultures is highly recommended. The specific surface treatment of these plates promotes optimal adhesion of Matrigel drops, ensuring stable dome formation for organoid growth. Plates from other manufacturers may result in Matrigel to spread across the well surface, limiting spheroid and organoid three‐dimensional expansion.

1. Allow the plate to sit at room temperature for 2–3 min, then invert it upside down.
2. Incubate at 37°C with 5% CO_2_ for at least 20 min, and up to a maximum of 45 min, until the Matrigel has polymerized.
3. Add the appropriate volume of complete Minigut medium to each well to obtain human intestinal organoids (Figure 3vii).

**Colonic regionalization (Day 9–12)**

To obtain HCOs, follow Steps 40–49 for spheroid embedding, but use complete Minigut B medium during Step 49. Then, proceed with Step 50 as usual, switching to complete Minigut medium without BMP2.

**Maintenance of organoids (Day 9–37)**

1. Renew the complete Minigut medium every 4 days during 28 days.

**Passaging of organoids (Day 23)** (Figure 3viii)

**NOTE**: In this protocol, we opted for mechanical dissociation as a gentler approach that better preserves tissue integrity and maintains the desired mesenchymal content, which is often difficult to control with enzymatic dissociation. Moreover, this method enables the visual selection of organoids under a stereomicroscope based on their morphology, enabling the exclusion of poorly structured aggregates. However, mechanical dissociation has certain limitations, including incomplete removal of residual Matrigel and dead cells during microdissection.

1. Thaw the aliquots of Matrigel GFR on ice at least 2 h before plating.
2. Remove the old drops of matrigel containing the organoids and collect them in a petri dish using a sterile 1000 µL truncated tip.
3. Using microdissection tools and stereoscopic microscope, remove the residual matrigel, separate individual organoids, and cut larger ones in half if necessary.
4. Collect the organoids in a 15 mL conical tube using a sterile truncated 1000 µL tip and allow them to sediment. Carefully aspirate the supernatant.

**NOTE**: You can stack two sterile 1000 µL tips together on the aspiration system to reach the bottom of the tube and aspirate without tilting it.

1. Depending on the number of organoids, add an appropriate volume of cold GFR Matrigel (e.g., 30–40 µL per number of drops required).

**▲CRITICAL**. Always handle Matrigel on ice to prevent premature polymerization.

1. Mix thoroughly but avoid creating bubbles. Deposit 30 to 40 µL drops and homogenize between each drop. **NOTE**: Regularly place the tube back on ice to prevent premature polymerization.
2. Allow the plate to sit at room temperature for 2–3 min, then invert it.
3. Incubate at 37°C with 5% CO_2_ for at least 20 min, up to a maximum of 45 min, until the Matrigel is fully polymerize.
4. Add the appropriate volume of complete Minigut medium to each well.

**Stopping organoids culture (Day 37)** (Figure 3ix)

**FIGURE 4:**
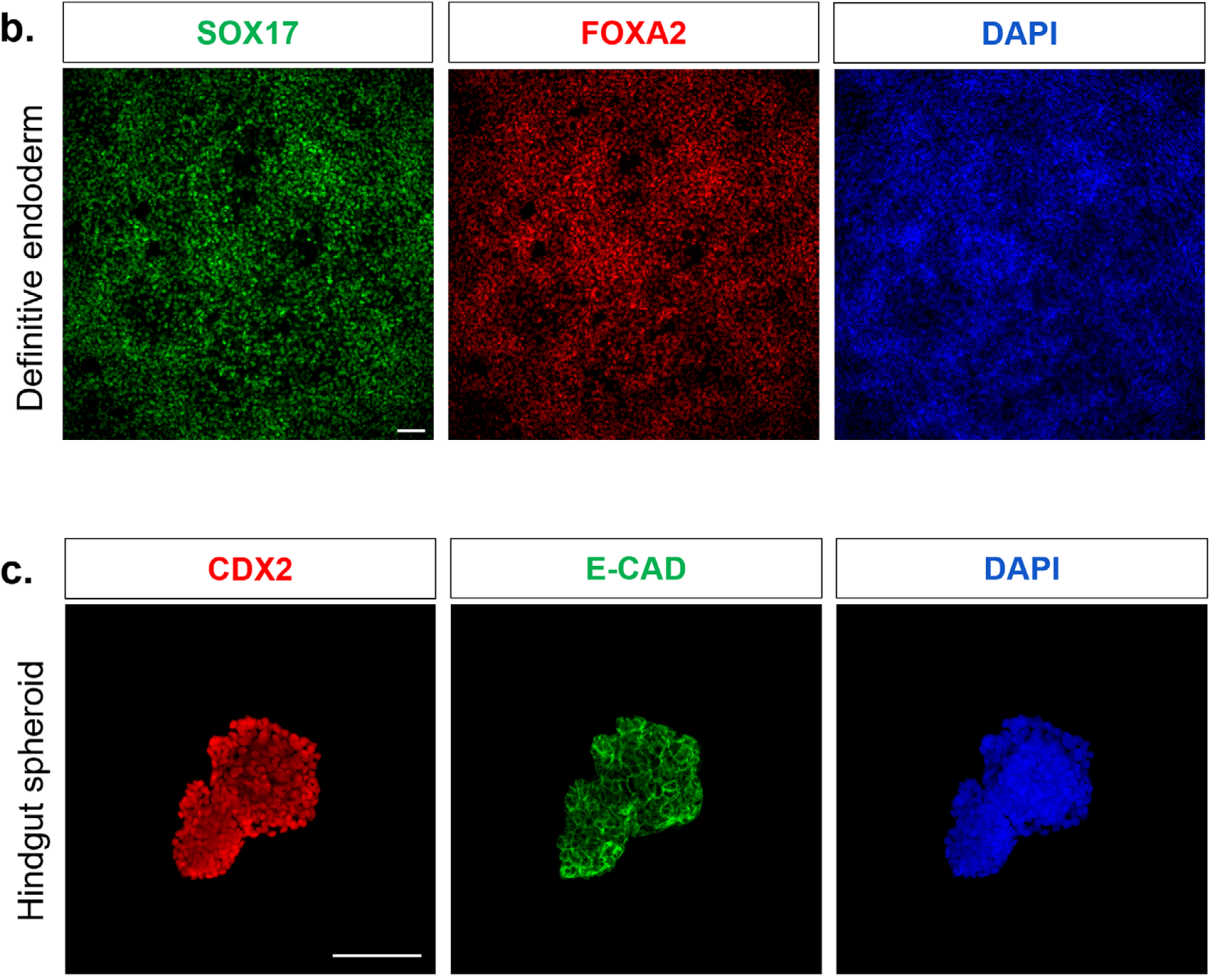
Characterization of definitive endoderm induction and spheroids generation. (a) Immunofluorescence images for FOXA2 and SOX17 of definitive endoderm after 3 days of Activin A induction. (b) Immunofluorescence images of hindgut spheroids following 4 days of FGF4 and Chiron induction, characterized by CDX2 (intestinal marker) and E‐cadherin (epithelial marker) expression. Scale bars = 100 µm.

**FIGURE 5:**
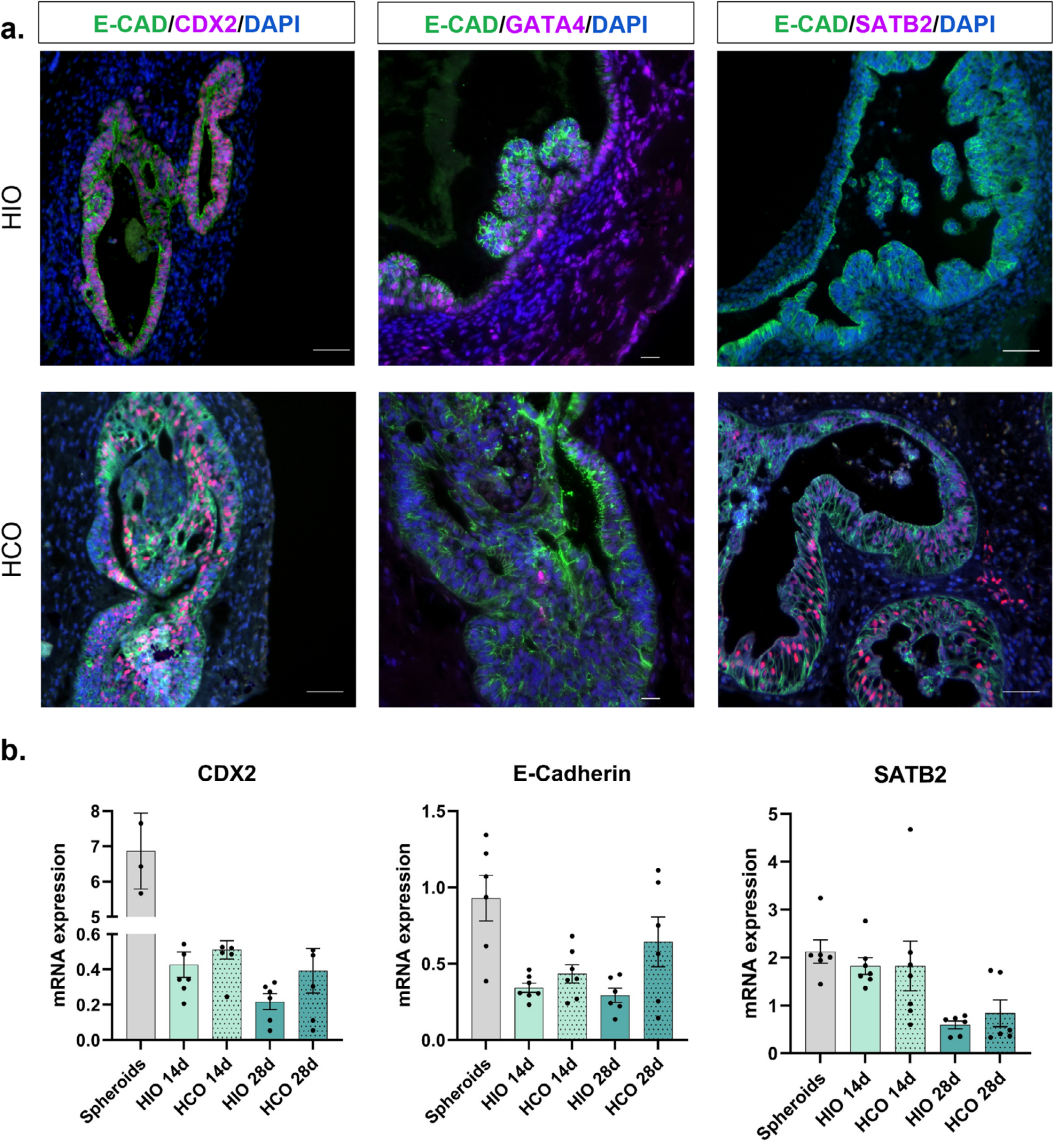
Characterization of hPSC‐derived HIO and HCO. (a) Immunofluorescence images of HIO and HCO sections stained for E‐cadherin (epithelial marker), CDX2 (intestinal marker), GATA4 (small intestinal marker), and SATB2 (colon marker) at day 37. Scale bars = 50 µm. (b) Relative mRNA expression levels of CDX2, E‐cadherin, and SATB2 were quantified by RT‐qPCR, and normalized to ACTB gene expression in spheroids, HIO, and HCO at days 14 and 28. Statistical analysis was performed using the Kruskal‐Wallis test (no significant differences observed).

1. For immunostaining, fix the organoids directly within Matrigel drops using 2% paraformaldehyde for 1 h and 30 min. Perform three washes with PBS containing 0.75% glycine. (Figures 4, 5)
2. For gene expression analyses, lyse organoids directly within the Matrigel drops using the lysis buffer appropriate for your extraction kit. (Figure 5)

## 5 Anticipated Results

## 6 Troubleshooting

**Table:** 

Step	Problem	Possible reason	Solution
Day 1	hPSCs are not dissociated into single cells.	Cells were not exposed to Accutase for a sufficient duration.	Increase the incubation time of Accutase and perform additional gentle pipetting to aid dissociation.
Day 1	Confluency of hPSCs is below 70%–80%.	The previous passaging of hPSCs was performed too early, insufficient cells were collected during maintenance, excessive cell death occurred during passaging.	Increase the passaging ratio, or anticipate this problem by preparing four wells of hPSCs instead of two to ensure adequate number of cells for differentiation.
Day 2–4	The monolayer is dying.	Dead cells generated during endoderm transition were not properly removed.	Gently shake the plate to resuspend and aspirate, or wash the layer with PBS (+/+) before adding fresh medium.
Day 9	There are no spheroids in the plates.	Too few cells attached when seeding at Day 0, or dead cells were not adequately removed.	Use vital dye when counting cells to increase accuracy. Mix thoroughly when resuspending cells and preparing the counting solution. Check the quality of the Matrigel batch.
Day 9–37	Spheroids do not survive in GFR matrigel drops.	Spheroids were too small at the time of embedding.	Repeat the experiment, being more vigilant regarding the aforementioned steps. Consider increasing the seeding cell density (test 3–4 densities between 53,000 cells cm^−2^ and 79,000 cells cm^−2^).

## Conflicts of Interest

The authors declare no conflicts of interest.

## Data Availability

The data that support the findings of this study are available from the corresponding author upon reasonable request.
